# Antimicrobial activity of polyphenol-rich fractions from *Sida alba *L. (Malvaceae) against co-trimoxazol-resistant bacteria strains

**DOI:** 10.1186/1476-0711-11-5

**Published:** 2012-02-24

**Authors:** Kiessoun Konaté, Adama Hilou, Jacques François Mavoungou, Alexis Nicaise Lepengué, Alain Souza, Nicolas Barro, Jacques Y Datté, Bertrand M'Batchi, Odile Germaine Nacoulma

**Affiliations:** 1 Laboratoire de Biochimie et Chimie Appliquées (LABIOCA), UFR/SVT, Université de Ouagadougou, 09 BP 848 Ouagadougou 09, Burkina Faso; 2 Institut de Recherche en Ecologie Tropicale (IRET/CENAREST), BP: 13354 Libreville, Gabon; 3 Laboratoire de Phytopathologie, UFR Agrobiologie, Faculté des Sciences, Université des Sciences et Techniques de Masuku, BP 943 Franceville, Gabon; 4 Laboratoire de Physiologie Animale, Electrophysiologie et de Pharmacologie, Faculté des Sciences, Université des Sciences et Techniques de Masuku, Franceville, Gabon; 5 Laboratoire de Biochimie et de Génétique moléculaire microbienne, UFR/SVT, Université de Ouagadougou, 03 BP 7131 Ouagadougou 03, Burkina Faso; 6 Laboratoire de Nutrition et de Pharmacologie, UFR BioSciences, Université de Cocody, Abidjan 22 BP: 582 Abidjan 22, Côte d'Ivoire

## Abstract

**Background:**

The increased resistance of microorganisms to the currently used antimicrobials has lead to the evaluation of other agents that might have antimicrobial activity. Medicinal plants are sources of phytochemicals which are able to initiate different biological activities including antimicrobials

**Materials and methods:**

*In vitro *antibacterial (MIC, MBC and time-kill studies) of polyphenol-rich fractions from *Sida alba *L. (Malvaceae) was assessed using ten bacteria strains (Gram-negative and Gram-positive).

**Results:**

All test bacteria were susceptible to the polyphenol-rich fractions. Time-kill results showed that after 5 h exposition there was no viable microorganism in the initial inoculum and the effect of polyphenol-rich fractions was faster on *Enterococcus faecalis *(Gram-positive bacterium) comparatively to the other bacteria strains.

**Conclusion:**

The data analysis indicates that the tested of polyphenol-rich fractions has significant effects when compared with the standard antibiotic. These results therefore justify the traditional use of *sida alba *L., alone or in combination with other herbs to treat bacterial infections.

## Background

The use of medicinal herbs in traditional system of medicine is a common practice in many cultures around the world especially in African societies. This practice has gained widespread acceptance in developing as well as in developed nations. Researchers are also beginning to appreciate the role of medicinal plants in health care delivery [1]. In recent time, interest with herbal medicine for antimicrobial activities has been increased significantly. This is as a result of the effectiveness, low cost and the availability of these herbal medicines, the economic crisis, high cost of industrialized medicines, inefficient public access to medical and pharmaceutical care, in addition to the side effects caused by synthetic drugs are some of the factors contributing to the central role of medicinal plants in health care [2,3].

So, there is serious need to develop new antimicrobial agents that are very effective with minimal unwanted side effects and higher plants represent a potential source of novel antibiotic prototypes [4]. Medical plants have shown a promising alternative for the treatment of infectious diseases. In the antibacterial research, the vast majority, 78% of the new chemical entities are natural or natural products derived molecules [5].

In effect, at last 35,000 plant species are used for medicinal purposes throughout the world [6]. The most important industrial medicines nowadays are based on about 90 species of herbs and in developing countries, traditional remedies are usually based on mixtures of herbs collected from nature [7]. Among such plants, *Sida alba *L. (Malvaceae), is a herbaceous plant broadly distributed in tropical and subtropical areas. The medicaments are prepared most often from a combination of two or more plant products, which many a time many contain active constituents with multiple physiological activities and could be used in treating various disease conditions [7]. In Burkina Faso, the aqueous extracts of *Sida alba *L. (Malvaceae) are mostly used in popular folk medicine for the treatment of several diseases, particularly in the hepatitis virus diseases [8]. Ethnobotanical investigations in the central region of Burkina Faso and certain recent studies performed in laboratory have shown that *Sida alba *L. is frequently and widely used in traditional medicine to treat various kinds of diseases such as infectious diseases in children, malaria, fever, pain, variola, antibacterial, anti-inflammatory, analgesic activities and hepatoprotective [8,9]. Previous studies performed in our laboratory showed that aqueous acetone extract of *Sida alba *L. contains saponosides, coumarins, steroids, polyphenol compounds and alkaloids and possess antioxidant and ant-inflammatory properties [10]. Because pharmacological properties of *Sida alba *L. are highly attractive to assess for a new drug discovery, the present investigation reports the antimicrobial activities of polyphenol-rich fractions from *Sida alba *L. The bacteriostatic, bactericidal and time-kill assay studies of polyphenol-rich fractions were screened against clinical strains of bacteria. The aim was to justify the traditional antimicrobial use of this species and to produce scientific data for a future project for manufacturing phytomedicines, for use in combination with conventional antimicrobial drugs to better manage resistant bacteria infectious diseases.

## Materials

### Plants material

*Sida alba *L. was collected fresh in August 2008 in Gampela, 25 Km east of Ouagadougou, capital of Burkina Faso. The plant was botanically identified by Prof. Millogo-Rasolodimby from the plants Biology Department of the University of Ouagadougou. Voucher specimen (ID-10473) was deposited at the Herbarium of the "Laboratoire de Biologie et d'Ecologie Végétale, UFR/SVT of University of Ouagadougou".

### Bacterial strains and antibiotics

Microorganisms used in this study were isolated from clinical samples at Laboratory of the General Hospital of Ouagadougou in Burkina Faso. Commercially available antibiotic diffusion discs (10 μg/disc) and Co-trimoxazol were purchased from Alkom Laboratories LTD. Clinical isolates were: *Shigella dysenteriae, Shigella boydii, Shigella flexneri, Salmonella thyphi, Klebsiella pneumonia, Klebsiella arogenes, Escherichia coli, Enterococcus faecalis, Enterobacter aeruginosa *and *Proteus mirabilis*. The following microorganisms were all identified by the use of their biochemical profiles as recommended by the manual "Bactériologie Medical" [11].

### Chemicals

All reagents were of analytical grade. Acetone, n-hexane were supplied by Fluka chemie (Buchs, Switzerland). INT (p-iodonitrotetrazolium chloride) was purchased from sigma-Aldrich chemie (Steinheim, Germany).

## Methods

### Polyphenols extraction

The harvested plant materials fresh (broken into leaf stems) were dried in the laboratory at room temperature (20-25°C), afterwards samples were ground to pass a sieve of 0.3 mm. Polyphenols were extracted with aqueous acetone (80%, v/v). The extract was then washed with hexane to remove chlorophyll and other low molecular weight compounds. Acetone was evaporated and the extract was lyophilized and stored at 22°C prior to biological tests. For the tests, lyophilized sample was dissolved with 10% DMSO in water at the desired concentration [9].

### *In vitro *antibacterial activity

#### Preparation of inocula

The susceptibility tests were performed by Mueller Hinton agar-well diffusion method [12]. The bacterial strains grown on nutrient agar at 37°C for 18 h were suspended in a saline solution (0.9%, w/v)NaCl and adjusted to a turbidity of 0.5 Mac Farland standard **(**10^8 ^CFU/ml). To obtain the inocula, these suspensions were diluted 100 times in Muller Hinton broth to give 10^6 ^colony forming units (CFU)/ml [13].

##### Preparation of discs

The stock solutions of polyphenols from *Sida alba *L. was dissolved in 10% dimethylsulfoxide (DMSO) in water [14] at a final concentration of 100 μg/ml after a serial two-fold dilution. Each stock solution of polyphenol-rich fractions from *Sida alba *L. was sterilized by filtration through 0.22 μm sterilizing Millipore express filter. The sterile discs (6 mm) were impregnated with 10 μL of the sterile polyphenol-rich fractions. Negative controls were prepared using discs impregnated with 10% DMSO in water and commercially available antibiotic diffusion discs (Co-trimoxazol from Alkom Laboratories LTD) were used as positive reference standards (10 μg/disc) for all bacterial strains.

##### Disc-diffusion assay

Petri plates (9 cm) were prepared with 20 ml of a base layer of molten Mueller Hinton agar (DIFCO, Becton Dickinson, USA). Each Petri plate was inoculated with 15 μl of each bacterial suspension (10^6 ^CFU/ml). After drying in a sterile hood, 6 mm diameter discs soaked with 10 μl of the different polyphenol-rich fractions dilutions were placed on the agar. Discs containing Co-trimoxazol were used as positive controls and 10% DMSO was used as a negative control. The plates were incubated for 24 h at 37°C and at 44°C for *Escherichia coli *because this bacterium is thermo resistant. The diameters of the inhibition zones were evaluated in millimeters. The extract inducing inhibition zone ≥ 3 mm around disc were considered as antibacterial. All tests were performed in triplicate and the bacterial activity was expressed as the mean of inhibition diameters (mm) produced [15].

##### Micro-well dilution assay

Minimum inhibitory concentration (MIC) was determined by the microdilution method in culture broth as recommended by [16]. Eight serial two-fold dilutions of polyphenol-rich fractions or conventional antibiotic were prepared as described before, to obtain final concentration range of 400 to 3.125 μg/ml. The 96-well micro-plates (NUNC, Danemark) containing 100 μL of Mueller Hinton (MH) broth were used. For each bacteria strain, three columns of eight wells to the micro-plate were used. Each well has getting: the culture medium + polyphenol-rich fractions or the combination of polyphenol-rich fractions with Co-trimoxazol + inoculum (10 μl of inocula) and INT (50 μl; 0.2 mg/ml). The plates were covered and incubated at 37°C and at 44°C for *Escherichia coli *for 24 h. All tests were performed in triplicate and the bacterial activity was expressed as the mean of inhibitions produced. Inhibition of bacterial growth was judged by rose or yellow colour. The MIC was defined as the lowest concentration of extract or fraction of extract at which no colony was observed after incubation. So, the MIC was defined as the lowest concentration at which no visible growth was observed.

##### Minimal bactericidal concentration (MBC)

Minimum bactericidal concentration (MBC) was recorded as a lowest extract concentration killing 99.9% of the bacterial inocula after 24 h incubation at 37°C. Each experiment was repeated at least three times. MBC values were determined by removing 100 μl of bacterial suspension from subculture demonstrating no visible growth and inoculating nutrient agar plates. Plates were incubated at 37°C for a total period of 24 h. The MBC is determined with the wells whose the concentrations are ≥ MIC [15,17]. The MBC were determined in Mueller Hinton (MH) agar (DIFCO, Becton Dickinson, USA) medium.

##### Evaluation of bactericidal and bacteriostatic capacity

The action of an antibacterial on the bacterial strains can be characterized with two parameters such as Minimum inhibitory concentration (MIC) and Minimum bactericidal concentration (MBC). According to the ratio MBC/MIC, we appreciated antibacterial activity. If the ratio MBC/MIC = 1 or 2, the effect was considered as bactericidal but if the ratio MBC/MIC = 4 or 16, the effect was defined as bacteriostatic [18].

##### Time-kill assay

A bactericidal effect is defined as a 3 Log decrease in the CFU/ml or a 99.9% kill over a specified time [19]. The definition of kill for this study has been used as per [20]. Kill-time can be determined at 6 h [21]. A 90% kill at 6 h is equivalent to a 99.9% kill at 24 h [22]. In this study the kill measurement was determined by the actual reduction in viable counts at 6 h for each isolate. Bacteria strains possessing the bactericidal effect were chosen to perform time-kill assay. Thus, 0.5 Mac Farland standards suspensions of the microorganisms were diluted to have 50 ml of approximately 10^6 ^CFU/ml in nutriment broth, and the concentration corresponding to the best MIC, were respectively added to the corresponding culture. The cultures were incubated at 37°C. At 0, 1, 2, 3, 4, 5 and 6 h, an aliquot of 100 μl was removed and diluted with 10 ml sterile broth. The obtained suspension was used to inoculate 9 cm diameter Petri plates with a sterile non toxic cotton swab on a wooden applicator as indicated before in the agar-well diffusion assay. After 24 h incubation at 37°C, the viability of the microorganisms was evaluated by the presence of colonies on the plates. The experiment was carried out twice following [9] method with light modifications.

## Results

In this present study, ten bacteria strain (Gram-negative and Gram-positive bacteria) were used. The antibacterial assays were performed by the agar-well diffusion and the broth micro dilution methods; so that they could be qualified and quantified by inhibition zone diameters, MIC, MBC, Time-kill assays. One noticed that the susceptibility of the bacteria to the polyphenol-rich fractions on the basis of inhibition zone diameters varied according to the microorganism, the results are reported in Table 1. There is a significant variation in the diameters of inhibition zone values (DIZ) of polyphenol-rich fractions (Table 1).

**Table 1:** Inhibition zone diameters (mm) recorder in agar-well diffusion assay using polyphenol-rich fractions from *Sida alba *L. and Co-trimoxazol

Microorganisms	Co-trimoxazol	Polyphenol
***Shigella dysenteria***	19.00 ± 1.73	22.66 ± 0.58
		
***Shigella boydii***	20.66 ± 0.58	21.33 ± 4.04
		
***Shigella flexneri***	19.00 ± 1.00	23.66 ± 4.93
		
***Salmonella thyphi***	nd	24.33 ± 1.15
		
***Klebsiella pneumonia***	nd	29.66 ± 1.53
		
***Klebsiella arogenes***	nd	14.00 ± 1.00
		
***Escherichia coli***	nd	29.00 ± 1.00
		
***Enterococcus faecalis***	21.00 ± 1.00	28.66 ± 1.53
		
***Enterobacter aeruginosa***	nd	22.00 ± 2.65
		
***Proteus mirabilis***	20.66 ± 1.53	24.66 ± 1.53

The results are the means of number of the colonies ± standard deviations. nd: no detected activity.

According to the results, the combination of ployphenol-rich fractions with Co-trimoxazol has the high activity comparatively to the polyphenol-rich fractions alone (Table 2).

**Table 2:** Inhibition zone diameters (mm) recorder in agar-well diffusion assay using the combination of polyphenol-rich fractions with Co-trimoxazol

*Microorganisms*	Polyphenol + Co-tri
***Shigella dysenteria***	27.66 ± 1.53

***Shigella boydii***	29.33 ± 0.58

***Shigella flexneri***	29.00 ± 1.73

***Salmonella thyphi***	12.33 ± 1.15

***Klebsiella pneumonia***	13.33 ± 2.31

***Klebsiella arogenes***	12.66 ± 3.79

***Escherichia coli***	12.33 ± 3.22

***Enterococcus faecalis***	29.66 ± 2.89

***Enterobacter aeruginosa***	14.66 ± 1.53

***Proteus mirabilis***	27.00 ± 1.00

The results are the means of number of the colonies ± standard deviations.

nd: no detected activity, Co-tri: Co-trimoxazol

As for the micro-well dilution assay (MIC) and Minimum bactericidal concentration (MBC) of polyphenol-rich fractions, result varied according to the microorganism (Table 3). About the micro-well dilution assay (MIC) and Minimum bactericidal concentration (MBC) of the combination of polyphenol-rich fractions with Co-trimoxazol, results were represented by (Table 4). The MIC values were ranged from 12.5 to 50 μg/ml and for the MBC values were ranged from 25 to 200 μg/ml. The bactericidal and bacteriostatic effect of polyphenol-rich fractions and the combination of polyphenol-rich fractions with Co-trimoxazol was determined using the ratio MBC/MIC (Table 3 and Table 4).

**Table 3:** Bacteriostatic (-) and Bactericidal (+) effects of *Sida alba *L. polyphenol-rich fractions

Microorganisms	MIC (μg/ml)	MBC(μg/ml)	Effects
***Shigella dysenteriae***	50 ± 00	100 ± 0.00	+

***Shigella boydii***	50 ± 0.00	100 ± 0.00	+

***Shigella flexneri***	100 ± 0.00	200 ± 0.00	-

***Salmonella thyphi***	100 ± 0.00	400 ± 0.00	-

***Klebsiella arogenes***	100 ± 0.00	400 ± 0.00	-

***Klebsiella pneumonia***	50 ± 0.00	200 ± 0.00	-

***Escherichia coli***	50 ± 0.00	200 ± 0.00	-

***Enterococcus faecalis***	25 ± 0.00	50 ± 0.00	+

***Enterobacter aeruginosa***	25 ± 0.00	100 ± 0.00	-

***Proteus mirabilis***	25 ± 0.00	50 ± 0.00	+

The results are the means of number of the colonies ± standard deviations.

**+: **bactericidal effect, - bacteriostatic effect

**Table 4:** Bacteriostatic (-) and Bactericidal (+) effects of combination of polyphenol-rich fractions with Co-trimoxazol

Microorganisms	MIC (μg/ml)	MBC (μg/ml)	MBC/MIC	Effect
***Shigella dysenteria***	25 ± 0.00	50 ± 0.00	2	+

***Shigella boydii***	25 ± 0.00	50 ± 0.00	2	+

***Shigella flexneri***	25 ± 0.00	50 ± 0.00	2	+

***Salmonella thyphi***	25 ± 0.00	200 ± 0.00	4	-

***Klebsiella pneumonia***	100 ± 0.00	400 ± 0.00	4	-

***Klebsiella arogenes***	100 ± 0.00	400 ± 0.00	4	-

***Escherichia coli***	100 ± 0.00	400 ± 0.00	4	-

***Enterococcus faecalis***	12.5 ± 0.00	25 ± 0.00	2	+

***Enterobacter aeruginosa***	100 ± 0.00	400 ± 0.00	4	-

***Proteus mirabilis***	12.5 ± 0.00	25 ± 0.00	2	+

The results are the means of number of the colonies ± standard deviations

**+: **bactericidal effect, -: bacteriostatic effect

Concerning the time-kill assay of polyphenol-rich fractions (Table 5) and the combination of polyphenol-rich fractions with Co-trimoxazol (Table 6), the results showed that after 5 h exposition there was no viable microorganism in the initial inoculums. The effect of polyphenol-rich fractions and the combination of polyphenol-rich fractions with Co-trimoxazol was faster on *Enterococcus faecalis *than the other bacteria strains (Table 5 and Table 6).

**Table 5:** Viability of microorganisms after 6 h exposure of polyphenol-rich fractions from *Sida alba *L

Microorganisms			Time-kill (h)				
	**0 h**	**1 h**	**2 h**	**3 h**	**4 h**	**5 h**	**6 h**

***Shigella dysenteriae***	+ (UC)	+ (UC)	23 ± 03	09 ± 02	04 ± 02	-	-
***Shigella boydii***	+ (UC)	+ (UC)	25 ± 02	08 ± 02	05 ± 02	-	-
***Enterococcus faecalis***	+ (UC)	+ (UC)	21 ± 01	08 ± 03	-	-	-
***Proteus mirabilis***	+ (UC)	+ (UC)	17 ± 0.00	05 ± 01	-	-	-

The results are the means of number of the colonies ± standard deviations.

+: For the presence of the colonies

-: for absence of colonies

UC: uncountable

**Table 6:** Viability of microorganisms after 6 h exposure of the combination of polyphenol extracts with Co-trimoxazol

Microorganisms			Time-kill (h)				
	**0 h**	**1 h**	**2 h**	**3 h**	**4 h**	**5 h**	**6 h**

***Shigella dysenteriae***	+ (UC)	+ (UC)	23 ± 03	14 ± 01	05 ± 03	-	-
***Shigella boydii***	+ (UC)	+ (UC)	25 ± 01	13 ± 01	04 ± 01	-	-
***Shigella flexneri***	+ (UC)	+ (UC)	23 ± 03	14 ± 02	05 ± 01	-	-
***Enterococcus faecalis***	+ (UC)	+ (UC)	21 ± 01	06 ± 01	-	-	-
***Proteus mirabilis***	+ (UC)	+ (UC)	26 ± 03	15 ± 01	07 ± 03	-	-

The results are the means of number of the colonies ± standard deviations.

+: For the presence of the colonies

-: for absence of colonies

UC: uncountable

## Discussion

Previous studies have been carried out in different parts of the globe to extract plant products for screening antibacterial activity [23]. Plants produce highly bioactive molecules that allow them to interact with other organisms in their environnement. Many of these substances are important in the defense against herbivores and contribute to the resistance to diseases [24]. Many investigators have evaluated the bioactivity of plant extracts and the isolated constituents against the serious infectious organisms [25].

In Africa, for the treatment of several infections, indigenous medicinal plants are often the only means [26]. Infectious due to multidrug-resistant microorganisms, pose an important clinical problem. Many of bacterial strains are resistant to the standard antibiotic (Co-trimozaxol) comparatively to the polyphenol-rich fractions. One could say that, the metabolites have been shown to be responsible for therapeutic activity of plants [27]. According to a recent study, aqueous acetone extract of *Sida alba *L. contains saponosides, coumarins, steroids, polyphenol compounds and alkaloids [10]. The natural products were found to possess promising antimicrobial activities when applied alone or in combination with conventional antimicrobial drugs and the metabolites have been shown to be responsible for therapeutic activity of plants [28]. This aspect is effectively checked by our different results.

The data analysis indicates that the tested polyphenol extract showed the significant results when compared with the Co-trimoxazol. Indeed, the antibacterial activity profile of the isolated constituents (polyphenols) when compared with antibiotic effects shows that the activity depends on the pure form of the constituents. This may be due to the fact that the bioactive constituents such as polyphenol compounds were responsible for the antimicrobial activity. In effect, some previous studies showed that polyphenolic compounds cause inhibition of a wide range of microorganisms. Phenol is well known as a chemical antiseptic [29]. In addition, Phenolic and terpenic antimicrobial activities are well documented [15]. Polyphenols, such as tannins and flavonoids, are important antibacterial activity [26]. The antimicrobial activity of flavonoids is due to their ability to complex with extracellular and soluble protein and to complex with bacterial cell wall while that of tannins may be related to their ability to inactivate microbial adhesions, enzymes and cell envelop proteins [24]. We noticed that our extracts or in combination showed relatively the best inhibitory activity against *Enterococcus faecalis *a Gram-positive bacterium followed by *Proteus mirabilis *a Gram-negative bacterium. The highest sensitivity of *Enterococcus faecalis *may be due to its cell wall structure and outer membrane [30]. Gram-positive bacteria are generally more sensitive in the extracts than Gram-negative bacteria [31]. A possible explanation for these observations may lie in the significant differences in the outer layers of Gram-positive bacteria. The permeability of the cell wall of the Gram-negative organism is generally less efficient than Gram-positive ones probably because of the presence of the high level of phospholipids in the cell wall compared with Gram-positive bacteria [32].

Gram-negative bacteria however, possess an outer membrane and unique periplasme space not found in Gram-positive bacteria [32]. The resistance of Gram-negative bacteria towards antibacterial substances is related to the hydrophilic surface of their outer membrane which is rich in lipopolysaccharide molecules, presenting a barrier to the penetration of numerous antibiotic molecules and is also associated with the enzymes in periplasme space, which are capable of breaking down the molecules introduced from outside [33]. The resistances of the bacteria to the current antibiotics necessitate the further studies on the isolated constituents to find out their safety and efficacy profile. This highlights the continuous interest in laboratory screening of medicinal plants, not only to determine the scientific rationale for their usage, but also to discover new active principles. African medicinal plants have been focused on phenolic compounds, terpenoids or essential oils [34]. The plants have been found to exert good *in vitro *antimicrobial activities and some active principles have been isolated.

## Conclusion

In short according our results, polyphenol-rich fractions from *Sida alba *L. were found to possess promising antimicrobial activities when applied alone or in combination with conventional antimicrobial drugs to treat infectious diseases due to multi-resistant bacterial strains.

## Competing interests

The authors declare that they have no competing interests.

## Authors' contributions

KK and AH carried out the study and wrote the manuscript. JMF, ANL, AS and NB supervised the work and the manuscript. JYD, BMB and OGN contributed to the manuscript corrections. All authors read and approved the final manuscript.
